# Prospective controlled study comparing patient-reported outcomes after daily online adaptive radiotherapy or conventional IGRT in patients with prostate cancer

**DOI:** 10.1016/j.ctro.2025.101092

**Published:** 2025-12-05

**Authors:** Goda Kalinauskaite, Luise A. Künzel, Kerstin Rubarth, Thao Nguyen, Jakob Dannehl, Celina Höhne, Marcus Beck, Julia Bauer, Daniel Zips, Carolin Senger

**Affiliations:** a Charité – Universitätsmedizin Berlin, Corporate Member of Freie Universität Berlin and Humboldt-Universität zu Berlin, Department of Radiation Oncology, Augustenburger Platz 1, 13353 Berlin, Germany; b Berlin Institute of Health at Charité – Universitätsmedizin Berlin, Charitéplatz 1, 10117 Berlin, Germany; c Charité Universitätsmedizin – Universitätsmedizin Berlin, Corporate Member of Freie Universität Berlin and Humboldt-Universität zu Berlin, Institute of Biometry and Clinical Epidemiology, Charitéplatz 1, 10117 Berlin, Germany; d German Cancer Research Center (DKFZ), Heidelberg and German Cancer Consortium (DKTK) Partner Site Berlin 10117 Berlin, Germany; e National Center of Tumor Diseases (NCT), Heidelberg, Germany

**Keywords:** Prostate cancer, Online adaptive radiotherapy, Cone-beam computed tomography, Image guided radiotherapy, Patient-reported outcome measures, Health-related quality of life

## Abstract

**Purpose:**

To compare patient-reported outcome measures (PROMs) in patients treated either with cone-beam CT (CBCT)-based online adaptive radiotherapy (oART) or with CBCT-guided conventional image guided radiotherapy (IGRT).

**Materials and methods:**

In this prospective study with convenience allocation, patients with localized prostate cancer received 62 Gy/20 fractions using either daily CBCT-based oART or CBCT-guided conventional IGRT. PROMs (EPIC, QLQ-PR25, IPSS, NCI-PRO-CTCAE) were collected at baseline and at end of therapy. Changes in scores and clinically meaningful deterioration, based on established minimal clinically important differences (MCID), were analyzed.

**Results:**

Seventy-four patients were included (oART: 58.1 %; IGRT: 41.9 %). Groups were demographically similar, although the oART group included more patients with high-risk tumors (40.5 % vs. 9.7 %, p = 0.03). Patients after oART tended to experience smaller, although not statistically significant, declines in health-related quality of life (HRQoL) domains compared to IGRT: EPIC urinary summary (−12.15 vs −20.57, p = 0.07), urinary function (−9.53 vs −17.47, p = 0.05), urinary incontinence (−5.47 vs −13.93, p = 0.07) and PR25 urinary symptom (20.0 vs. 27.5, p = 0.06). EPIC bowel function decline was also less pronounced (−12.64 vs. −19.78, p = 0.10). NCI-PRO-CTCAE scores favored oART for reduced urinary urgency (0.95 vs. 1.57, p = 0.02) and fecal incontinence (0.03 vs. 0.71, p = 0.02). Fewer oART patients reached MCID thresholds for urinary (8–21 %) and bowel (20–23 %) deterioration, but these differences were not statistically significant.

**Conclusion:**

Our results suggest a small but consistent trend in PROM scores favoring oART over conventional IGRT. In addition, the results may inform the design of controlled randomized trials in the future**.**

## Highlights

• Urinary HRQoL decline tends to be smaller with CBCT-based oART than IGRT.
• Patients treated with online ART show reduced urinary urgency post-treatment.
• Online ART reduced MCID in bowel symptoms by 20 % vs. IGRT.
• Online adaptation potentially enhances organ sparing without changing PTV margins.

## 1 Introduction

Improvements in radiotherapy have led to low toxicity rates in prostate cancer patients. Although rare, intermediate or high-grade toxicity can have a negative effect on health-related quality of life (HRQoL), as measured by patient-reported outcome measures (PROMs) [[1], [2], [3], [4]].

Technological advances—improved on-couch imaging quality, synthetic CT with accurate Hounsfield-unit representation, integration of artificial intelligence, and fast planning algorithms—have enabled the development of online adaptive radiotherapy (oART) [5,6]. Hybrid MR- or CBCT-guided linear accelerators allow plan adaptation immediately before each treatment session, potentially reducing toxicity through improved sparing of organs at risk (OAR) [[7], [8], [9], [10], [11], [12]]. In addition, oART may facilitate planning target margins reduction which may further enhance OAR sparing [13,14].

In the non-randomized MOMENTUM study using MR-guided ART, 146 patients with prostate cancer were treated with 60 Gy in/20 fractions [15]. Low rates of acute Grade ≥ 2 toxicity (GI 3 %, GU 7 %) three months post treatment were observed. Quality of life declined clinically significant within six months post-radiotherapy but recovered by 24 months. Importantly, prospective data comparing CBCT-based oART with conventional IGRT in this patient group is limited.

This prospective controlled study aims to compare acute outcomes, as measured by PROMs in prostate cancer patients treated either with CBCT-based oART or with CBCT-guided conventional IGRT.

## 2 Methods

### 2.1 Study design and study population

This prospective, single-institution, unblinded interventional study with convenience allocation was approved by the local ethics committee (EA1/123/23) and registered on ClinicalTrials.gov (NCT06116019). Patients who provided written informed consent were enrolled between January and November 2024. Inclusion criteria were: histologically confirmed localized prostate cancer of any National Comprehensive Cancer Network (NCCN) risk group [16]. Exclusion criteria included inability to remain motionless during the treatment, or inability to provide informed consent.

As prospectively defined, slots for oART were limited to a maximum of five per day. Allocation was determined solely by operational capacity: after consent for either oART or IGRT, patients were assigned to oART when one of the five oART treatment slots was available; otherwise, they received IGRT. All other patients received conventional IGRT, resulting in convenience allocation.

### 2.2 Intervention

Radiotherapy planning used a dedicated pelvic planning CT (2-mm slices; SOMATOM go.Open Pro, Siemens Healthineers, Erlangen, Germany) with patients supine and instructed to maintain bladder filling and empty rectum. Treatment comprised 20 fractions with a simultaneous integrated boost at three dose levels (62.0/57.6/48.0 Gy). Three CTVs were defined: CTV_62 (prostate expanded to include the involved part of the seminal vesicles [SV] in cT3b disease); CTV_57.6 (CTV_62 expanded by 0.5 mm in all directions [excluding the rectum] and including the base of SV in cT3b cases); CTV_48 (CTV_57.6 to encompass the distal 2 cm of the SV in cT1–3a or the entire SV in cT3b disease). Planning target volumes (PTV) corresponding to the respective CTV were generated by adding a 5/3 (posteriorly) mm margin. Planning goals for target coverage and OAR constraints are summarized in Supplementary Table S1. Intensity‐modulated radiotherapy was delivered using either nine or twelve fields. The same planning goals were applied to both treatment groups. No elective nodal irradiation was applied. Fiducial markers were not used in both groups.

All initial plans were measured using an array in a rotating cylindrical phantom (Octavius 4D, PTW Freiburg, Germany) and reconstructed 3D dose distributions were compared to calculated ones in a 3D gamma analysis (2 mm/3 % maximum dose). Plans were accepted for treatment if passing rate greater 95 % was reached. For on couch plan quality assurance during adaptive sessions an independent secondary dose calculation in Mobius3D (Varian Medical Systems) was used for 3D gamma analysis with same criteria as for initially measured plans.

Radiotherapy was administered five days per week using a CBCT-equipped linear accelerator capable of online adaptive radiotherapy (ETHOS™ Therapy System, Varian Medical Systems, Palo Alto, CA, USA). In the IGRT group, daily CBCT verification was performed, and any unacceptable deviations in bladder or rectal filling compared to the planning CT were corrected before initiating the treatment. In the oART group, corrections were made only if large rectal air pockets were present.

IGRT was delivered using CBCT followed by registration with planning CT. After assuring that the targets are covered by the respective PTVs, the patient position was accordingly corrected by table shift and irradiation was started. The oART and its delivery have been detailed elsewhere [17]. Briefly, oART was delivered daily by re-optimizing the initial plan based on the patient’s anatomy on daily CBCT, adhering to the constraints. A multidisciplinary team – including radiotherapy technologists, a medical physicist expert and a senior radiation oncologist – were involved in the treatment and were on site. The oART workflow consisted of the following steps: acquisition of the initial CBCT, definition and review of daily anatomy and targets, plan evaluation, plan quality assurance, and position-verification CBCT. An adaptive plan was selected at the discretion of the physician if it improved PTV coverage or provided better OAR sparing or both. If the position-verification CBCT showed unacceptable anatomical deviations, the entire workflow was repeated.

### 2.3 Data collection and statistical analysis

This prospective controlled study aimed to compare acute PROMs, which were collected using validated questionnaires at baseline (on the first day of radiotherapy) and at the end of treatment (during the final treatment days, fractions 19–20). (I) EORTC QLQ-C30, (II) QLQ-PR25, (III) EPIC (EPIC 2. 2002, 32 questions), (IV) IPSS, (V) FACIT-F, (VI) NCI-PRO-CTCAE (comprising 17 self-preselected questions, Table S2), and (VII) an adapted questionnaire on patient-reported treatment experience [18].

Changes from baseline to the end of therapy in patient-reported outcomes — assessing HRQoL with EPIC and PR25, and acute toxicity with NCI-PRO-CTCAE and IPSS — were compared between the IGRT and oART groups. Questionnaires were scored according to the respective manuals [19,20]. For the EPIC questionnaire, higher scores (0–100) indicate better HRQoL. In the QLQ-PR25, higher scores (0–100) on the functional scales indicate better function, whereas higher scores on the symptom scales reflect greater symptom severity. The NCI-PRO-CTCAE (0-4) and IPSS (0-35) also use scales in which higher values indicate more severe symptoms. In a second step, we assessed whether changes in QLQ-PR25 and EPIC between the treatment modalities were also clinically relevant.

Baseline characteristics were summarized using descriptive statistics (metric data were presented as means [standard deviations] and categorical data as absolute frequencies [relative frequencies]). Although the data were measured on a Likert scale, means and standard deviations were reported instead of medians and quartiles, as the former would allow for a better differentiation between groups, which might not have been as apparent with medians and quartiles. Furthermore, data were approximately symmetrically distributed. Differences between baseline characteristics of oART and IGRT groups were evaluated by using two-sided t-tests in case of continuous variables and Chi-square-tests in case of categorical variables. Changes in PROMs from baseline to the end of therapy were calculated for each domain and were tested by two-sided t-tests. Further, minimal clinically important differences (MCIDs) were retrieved from literature. For the QLQ-PR25, an MCID was defined as a change of at least 10 points, in accordance with the QLQ-C30 scoring manual [20]. For the EPIC questionnaire, the MCIDs were defined as the mean value of the MCID range defined for EPIC-26 with a change of 5 points for gastrointestinal function, a change of 7 points for urinary incontinence, a change of 11 points for sexual function and a change of 6 points for urinary symptoms [21]. The proportions of patients who experienced a clinically significant worsening were compared between the oART and IGRT groups by Chi-squared tests. A p-value of 5 % was considered statistically significant and no adjustment for multiplicity was conducted due to the exploratory nature of the study. As an additional sensitivity analysis, we repeated all primary analyses after excluding patients classified as high-risk (n = 20), given the imbalance in high-risk prevalence between treatment groups.

No formal sample size calculation was conducted due to the lack of pilot data and effect sizes. With a sample size of 74 patients (42 in the oART group and 31 in the other group), a standardized mean difference of Cohen’s d = 0.66 (moderate-to-large effect) can be detected with 80 % power at α = 0.05 (two-sided). Given the exploratory nature of the study, smaller effects are reported, acknowledging their potential clinical relevance and statistical uncertainty.

All statistical analyses were performed using R Version 4.4.1. To visualize the data, box plots were generated using the ggplot package (Version 3.5.2) to compare the distribution of change scores across treatment groups, highlighting medians, interquartile ranges, means. Individual patient data was also visualized in these plots.

## 3 Results

**Fig. 1:**
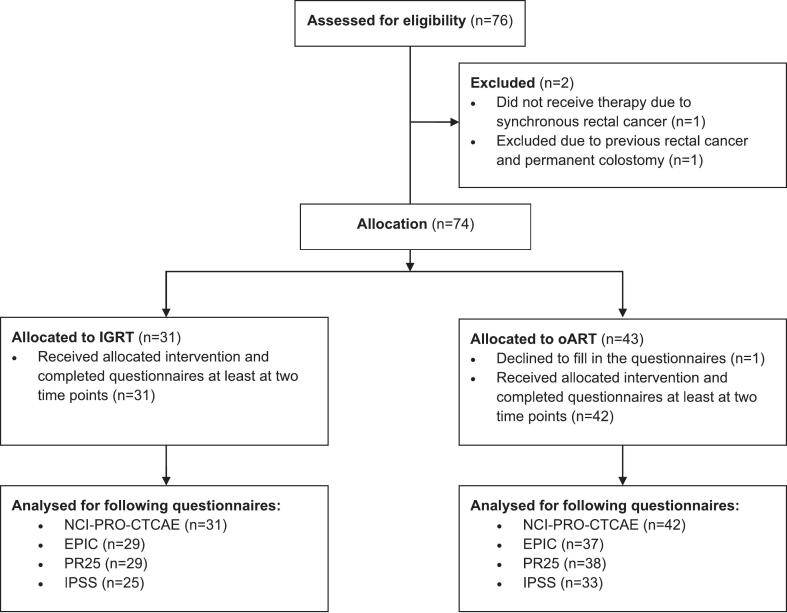
Study flow chart (IGRT: image guided radiotherapy; oART: online adaptive radiotherapy).

Seventy-six patients were screened and 74 patients were allocated to oART (n = 43; 58.1 %) or IGRT (n = 31; 41.9 %, Fig. 1). Two patients were excluded from the study: one due to simultaneous rectal carcinoma and the second due to previous rectal cancer with a permanent colostomy. In addition, one patient in the oART group declined to complete the questionnaires after treatment allocation. Baseline characteristics are listed in Table 1. Notably, the proportion of high-risk prostate cancer was higher in the oART group (40.5 %) compared to the IGRT group (9.7 %). However, other factors known to influence treatment-related toxicity, such as baseline urinary and bowel function and symptoms as measured with EPIC, IPSS and PR25 (Table S3a-c), prostate size (CTV volume), T stage, and dosimetric parameters including OAR constraints in the reference plans (Table S4), did not differ significantly between the groups. An adaptive plan was selected in 96 % of all fractions within the oART group.

**Table 1:** Patient characteristics.

Characteristic	ART (N = 42)	IGRT (N = 31)	p-value
Age, mean (SD)	72 (7.6)	72 (6.8)	0.92
Initial PSA, mean (SD)	10.54 (10.0)	8.09 (4.6)	0.21
NCCN risk group (%)			0.03
Low/Very low	3 (7.1)	4 (12.9)	
Favourable intermediate	17 (40.5)	16 (51.6)	
Unfavourable intermediate	5 (11.9)	8 (25.8)	
High/very high	17 (40.5)	3 (9.7)	
Clinical T-stage (%)			0.24
T1a-c	25 (59.5)	17 (54.8)	
T2a-c	10 (23.8)	12 (38.7)	
T3a-b	7 (16.7)	2 (6.5)	
CTV_62 vol (cm^3^), mean (SD)	59, 64 (17.92)	61.68 (18.59)	0.61
Androgen deprivation therapy (%)	18 (42.9)	10 (32.2)	0.50
Months from ADT initiation to the start of radiotherapy (mean, SD)	3.95 (5.48)	1.60 (1.58)	0.28
Changes in CTV_62 vol (cm^3^) during the therapy, mean (IQR)	−0.9 (−6.5 – 3.98)	N.A	

Abbreviations: ADT = androgen deprivation therapy; ART = adaptive radiotherapy; CTV = clinical target volume; IGRT = image-guided radiotherapy; IQR = interquartile range; N.A. = not applicable; NCCN = National Comprehensive Cancer Network; PSA = prostate-specific antigen; SD = standard deviation.

**Fig. 2:**
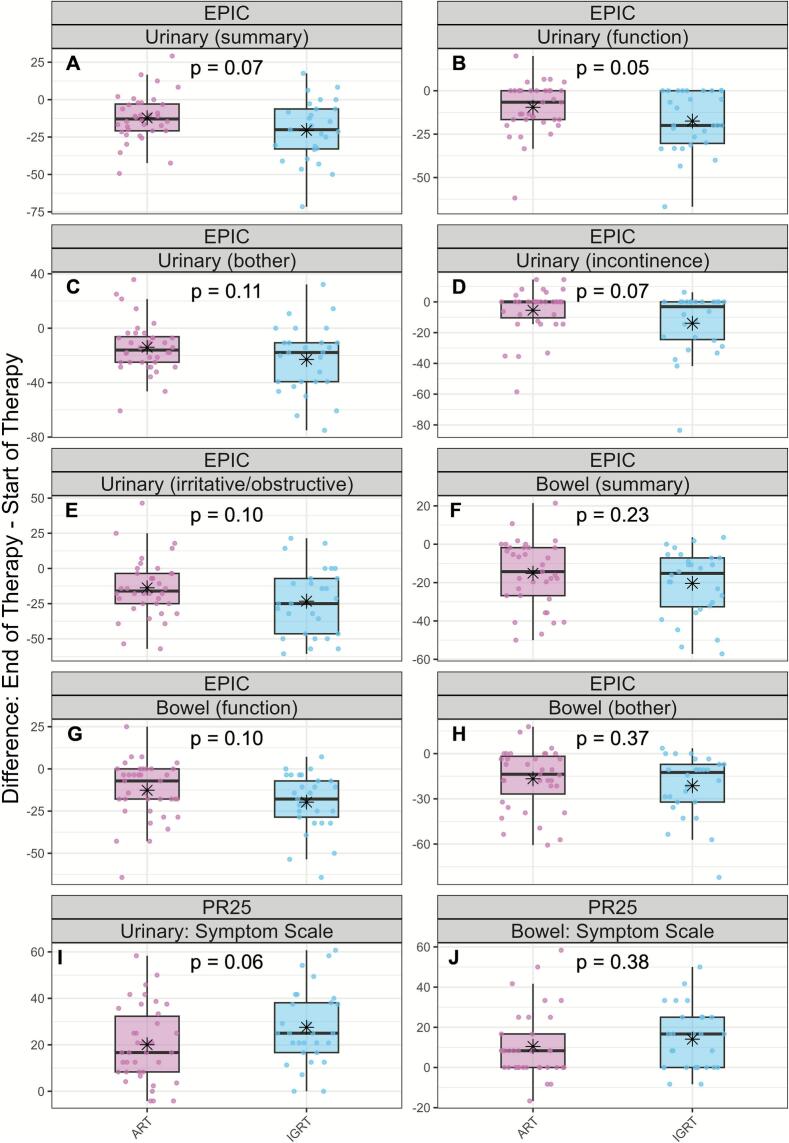
Changes in patient-reported urinary and bowel outcomes (oART vs. IGRT). Boxplots showing changes from baseline to end of therapy in urinary and bowel domains, assessed by EPIC (A–H) and PR25 (I, J). Light Red = oART; light blue = IGRT. Black asterisks represent group means. p-values for *t*-test. A higher EPIC score indicates a better outcome, whereas a lower PR25 score indicates a better outcome.

Compared to IGRT, patients treated with oART showed a not statistically significant trend towards less worsening in HRQoL, as measured by EPIC summary scores and domain-specific subscales at the end of therapy (p-values from 0.05 to 0.38). Fig. 2(A-H) shows box plots (light red oART, light blue IGRT) comparing changes from baseline to end of therapy for the urinary and bowel EPIC domains and subscales. For example, the urinary summary score decreased by mean (SD) 12.15 (16.60) in the oART group and 20.57 (20.50) in the IGRT group (p = 0.07; Fig. 2A, Supplementary Table S4 and S5). Bowel summary score for oART and IGRT decrease was 15.13 (17.23) and 20.41 (17.36) respectively (p = 0.23; Fig. 2G, Supplementary Table S5 and S6). Analysis of the EPIC sexual and hormonal domains revealed less worsening in the oART group compared to the IGRT group (Supplementary Tables S5 and S6).

Panels I and J in Fig. 2 display the PR25 urinary and bowel outcomes. The oART cohort showed a not statistically significant trend toward less symptom worsening than the IGRT cohort for both urinary (median [IQR]: oART 20.0 [16.0] vs IGRT 27.5 [16.1]; p = 0.06) and bowel symptoms (10.5 [16.7] vs 14.1 [15.4]; p = 0.38). Other PR25 symptom and functioning scales showed a small but consistently favourable differences in the oART group (Supplementary Table S7). Use of incontinence aids was very low (three in oART, none in IGRT); high missing-value rates made statistical testing infeasible.

**Fig. 3:**
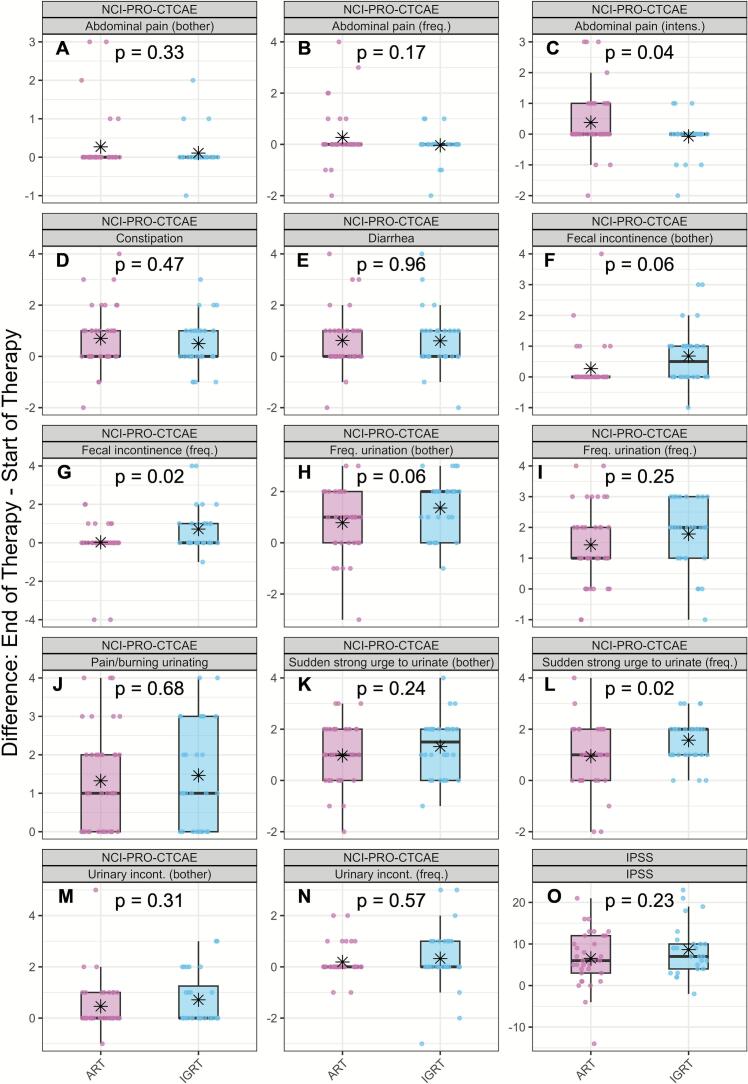
Changes in patient-reported acute toxicity (oART vs. IGRT). Boxplots showing changes from baseline to end of therapy in different domains, assessed by NCI-PRO-CTCAE (A–N) and IPSS (O). Light Red = oART; light blue = IGRT. Black asterisks represent group means. p-values for *t*-test. Lower scores on both the NCI PRO CTCAE and the IPSS indicate better outcomes.

NCI-PRO-CTCAE and IPSS assessments revealed few differences in acute toxicity profiles from baseline (Fig. 3, box plots in slight red oART, in light blue IGRT, Supplementary Tables S8 and S9). Except for intensity of abdominal pain (Fig. 3C), the difference to baseline scores for oART in most domains tended to be lower or equal (p-values ranging from 0.02 to 0.96), suggesting a trend toward fewer symptoms in oART patients compared with IGRT.

Across all EPIC summary scores and PR25 symptom scales, fewer oART patients tended to experience declines exceeding the MCID at the end of therapy; however, these differences were not statistically significant (p-values 0.077–0.652; Table 2). For example, in the PR25 urinary subdomain 26 out of 38 patients (68 %) experienced a clinical important decline in the oART group and 26/29 (89.7 %) in the IGRT group, i.e. 21.3 % less clinical important decline in the oART group (p = 0.077). Similarly, 23.1 % less clinical important decline for the bowel subdomain for oART versus IGRT.

**Table 2:** Minimal clinically important differences for EPIC and PR-25 questionnaires.

PROM	Subdomain	ART, n (%)	IGRT, n (%)	p-value
PR-25	Urinary	26/38 (68.4 %)	26/29 (89.7 %)	0.077
	Bowel	10/35 (28.6 %)	15/29 (51.7 %)	0.103
EPIC	Urinary incontinence	11/35 (31.4 %)	12/26 (46.2 %)	0.365
	Urinary irritative/obstructive	25/36 (69.4 %)	21/27 (77.8 %)	0.652
	Bowel	23/35 (65.7 %)	24/28 (85.7 %)	0.128

In the sensitivity analysis excluding high-risk patients, effect estimates were consistent with the main analysis and, in several outcomes, even more pronounced. Statistical significances were retained or strengthened (Supplementary Tables S10-S15 and Figs. S1/S2).

## 4 Discussion

To our knowledge, this is the first prospective study comparing CBCT-guided oART and conventional CBCT-guided IGRT focussing on PROMs in patients undergoing moderately hypofractionated radiotherapy for prostate cancer.

In summary, our findings indicate a consistent trend toward lower acute toxicity in the oART group compared with IGRT. Despite the small sample size and exploratory nature of the study, the results consistently, though mostly not statistically significantly, favoured oART across multiple urinary and bowel domains (Fig. 2, Fig. 3, Table 1). Importantly, these results were obtained using identical PTV margins in both groups.

Our IGRT cohort’s EPIC PROMs are consistent with those reported in previous studies [22,23]. In the study by Houben et al., urinary and bowel EPIC scores declined by 11.8 and 8.1 points after 70 Gy/28 fractions, whereas in our IGRT group, the corresponding declines were larger—20.5 and 20.4 points, respectively (difference 20.4, Supplementary Table S5) [22]. In the CHHIP trial (60 Gy in 20 fractions arm) lower differences in urinary and bowel scores 10 weeks after end of therapy were reported than in the IGRT arm in our trial [23]. The larger differences in our study likely reflect earlier assessment (week 4 of treatment) before post-therapy recovery seen in above mentioned trials.

The available evidence directly comparing adaptive and non-adaptive radiotherapy for prostate cancer remains limited [24,25]. A recent *meta*-analysis of 29 prospective studies (2,547 patients) compared acute toxicity between MR-guided adaptive and CT-guided non-adaptive SBRT. MR-guided adaptive SBRT was associated with lower rates of acute Grade ≥ 2 GU (16 % [95 % CI, 10 %–24 %] vs. 28 % [95 % CI, 23 %–33 %]) and GI (4 % [95 % CI, 2 %–7%] vs. 9 % [95 % CI, 6 %–12 %]) toxicities, despite similar PTV margins. However, the analysis included heterogeneous techniques and fractionation schemes, obscuring the specific impact of adaptation. In contrast, our study isolates the effect of online adaptation in a uniform, moderately hypofractionated CBCT setting.

Preliminary results from the international MOMENTUM registry (NCT04075305) showed that 1,373 prostate cancer patients treated with MR-guided adaptive radiotherapy (adapt-to-shape) had smaller increases in urinary symptom scores (PR25) at 3–12 months compared with non-adaptive treatment (adapt-to-position), while bowel and sexual outcomes were similar [24,25]. Our findings parallel these results—patients treated with CBCT-based oART reported smaller non-statistically significant urinary declines and fewer clinically meaningful deteriorations, suggesting that the potential benefits of daily adaptation may extend across adaptive therapy platforms and fractionation schemes. The MOMENTUM study also reported a transient increase in prostate volume (∼1.12× by the third fraction), which the authors discussed as a potential explanation for urinary differences. In our oART cohort, CTV_62 volume changes were minimal (mean −0.9 cc), likely reflecting moderate hypofractionation (20 fractions) and CBCT imaging, which offers lower soft-tissue contrast than MRI. The improved urinary outcomes observed in our study may reflect better bladder sparing, an aspect to be further explored in dose–, response analyses.

At the end of treatment, oART showed numerically less bowel symptom worsening than IGRT (EPIC bowel −15.1 vs −20.4; PR25 10.5 vs 14.1) and fewer patients exceeded MCID thresholds for deterioration—PR25 bowel (28.6 % vs 51.7 %) and EPIC bowel (65.7 % vs 85.7 %)—although these differences were not statistically significant. Fecal incontinence on NCI-PRO-CTCAE favored oART (0.03 vs 0.71; p = 0.02), while diarrhea and constipation were similar. Daily adaptation may enhance rectal sparing by accounting for interfractional variations of the rectum and seminal vesicles. This is consistent with a recent retrospective study (60 Gy in 20 fractions) that demonstrated rectal-dose benefits, with 48 % of fractions showing at least one clinically beneficial change (approximately 20 % related to the rectum). CBCT-guided oART was also associated with reduced clinician-graded GI toxicity compared with IGRT (OR 0.46; 95 % CI 0.22–0.92; p = 0.03) [26]. However, the oART group was treated with smaller PTV margins (4 mm vs 5 mm) and received hydrogel spacers more frequently (58 % vs 40 %) than the IGRT group, warranting cautious interpretation of the results. In our PROM-based analysis, we similarly observed less worsening of bowel symptoms with oART, suggesting a non-statistically significant trend toward improved rectal tolerance that should be confirmed in larger prospective studies.

Our study is limited by its convenience allocation, single-center design, and lack of blinding, all of which carry a risk of selection and reporting bias. Because allocation to oART was based on daily slot availability, complete randomization was not feasible, and patients were therefore assigned according to logistical rather than clinical criteria. This approach may have resulted in an uneven distribution of risk groups, as reflected by the higher proportion of high-risk tumors in the oART group (40.5 % vs. 9.7 %) and, although not statistically significant, a longer duration of ADT before the start of radiotherapy. Such an imbalance could potentially confound the results. However, other baseline factors known to influence treatment-related toxicity, including urinary and bowel function, prostate volume, T stage, and dosimetric parameters, were comparable between groups. As high-risk disease is generally associated with more extensive tumor burden and potentially greater treatment-related toxicity, this imbalance would, if anything, be expected to disadvantage the oART cohort. Nevertheless, the oART group reported lower acute urinary and bowel symptoms, suggesting that the observed benefits are unlikely to be explained by baseline risk differences alone. Moreover, the sensitivity analysis excluding all high-risk patients showed that the observed effects persisted and were even more pronounced in some outcomes. This indicates that the group imbalance in risk category did not substantially bias the results. The lack of blinding of both patients and clinicians represents an additional limitation. Awareness of the treatment modality could have influenced subjective reporting of PROMs, particularly in favor of the more novel oART approach. Finally, because PROMs were collected only at baseline and at the end of radiotherapy, subacute symptom peaks occurring in the weeks after treatment may not be fully captured. Post-treatment PROM follow-up is already planned to address this limitation.

Our results might inform the design of randomized trials. Based on differences observed in our EPIC subdomains, we performed sample size calculations using two‐sided t‐tests with a significance level α of 0.05 and 80 % power. In the domains with moderate or large effect sizes (e.g., urinary function [Cohen’s d = 0.46], sexual summary [Cohen’s d = 0.59]), at least 75 patients per group for urinary function or 46 patients per group for sexual summary would be required to detect clinically relevant differences. In contrast, domains with smaller effect sizes (e.g., bowel summary [Cohen’s d = 0.31]) would necessitate substantially larger cohorts, e.g. at least 165 patients per group for bowel summary. These calculations show that effect sizes, and thus the required sample size, depend on the hypothesis and the research domain.

## 5 Conclusion

Our results suggest a small but consistent trend in PROM scores favoring oART over conventional IGRT. This may suggest that daily oART could potentially enhance organ-at-risk sparing and functional outcomes, but confirmation in larger randomized trials is required. In addition, the data from this study could inform sample size calculations for future randomized trials using PROMs to further explore the potential benefit of oART.

## Funding statement

Goda Kalinauskaite is participant in the BIH Charité Digital Clinician Scientist Program funded the DFG, by the Charité – Universitätsmedizin Berlin, and the Berlin Institute of Health at Charité.

## Data sharing statement

The data used and generated in this work are available upon reasonable request from the lead institutions on an individual basis, providing that ethical and data protection considerations are met.

## CRediT authorship contribution statement

**Goda Kalinauskaite:** Conceptualization, Data curation, Investigation, Methodology, Project administration, Supervision, Funding acquisition, Resources, Writing – original draft. **Luise A. Künzel:** Conceptualization, Methodology, Project administration, Software, Resources, Writing – review & editing. **Kerstin Rubarth:** Formal analysis, Resources, Methodology, Visualization, Software, Writing – review & editing. **Thao Nguyen:** Formal analysis, Visualization, Software, Writing – review & editing. **Jakob Dannehl:** Data curation, Resources, Writing – review & editing. **Celina Höhne:** Data curation, Resources, Writing – review & editing. **Marcus Beck:** Investigation, Resources, Writing – review & editing. **Julia Bauer:** Writing – review & editing. **Daniel Zips:** Conceptualization, Investigation, Methodology, Project administration, Supervision, Funding acquisition, Writing – review & editing. **Carolin Senger:** Conceptualization, Investigation, Methodology, Project administration, Supervision, Funding acquisition, Resources, Writing – review & editing.

## Declaration of Competing Interest

The authors declare the following financial interests/personal relationships which may be considered as potential competing interests: [Daniel Zips reports a relationship with Varian Medical Systems Inc that includes: funding grants and speaking and lecture fees. If there are other authors, they declare that they have no known competing financial interests or personal relationships that could have appeared to influence the work reported in this paper.].

## Appendix A Supplementary data

Supplementary Fig. S1 Changes in patient-reported urinary and bowel outcomes (oART vs. IGRT) analyzed in the subgroup without high/very-high NCCN risk. Boxplots showing changes from baseline to end of therapy in urinary and bowel domains, assessed by EPIC (A–H) and PR25 (I, J). Light Red = oART; light blue = IGRT. Black asterisks represent group means. p-values for t-test. A higher EPIC score indicates a better outcome, whereas a lower PR25 score indicates a better outcome.

Supplementary Fig. S2 Changes in patient-reported acute toxicity (oART vs. IGRT) analyzed in the subgroup without high/very-high NCCN risk. Boxplots showing changes from baseline to end of therapy in different domains, assessed by NCI-PRO-CTCAE (A–N) and IPSS (O). Light Red = oART; light blue = IGRT. Black asterisks represent group means. p-values for t-test. Lower scores on both the NCI PRO CTCAE and the IPSS indicate better outcomes.

## References

[1] Kishan A.U., Ma T.M., Lamb J.M., Casado M., Wilhalme H., Low D.A. (2023). Magnetic resonance imaging–guided vs computed tomography–guided stereotactic body radiotherapy for prostate cancer: the MIRAGE randomized clinical trial. JAMA Oncol.

[2] Nikitas J., Jamshidian P., Tree A.C., Hall E., Dearnaley D., Michalski J.M. (2025). The interplay between acute and late toxicity among patients receiving prostate radiotherapy: an individual patient data meta-analysis of six randomised trials. Lancet Oncol.

[3] Kerkmeijer L.G.W., Groen V.H., Pos F.J., Haustermans K., Monninkhof E.M., Smeenk R.J. (2021). Focal boost to the intraprostatic tumor in external beam radiotherapy for patients with localized prostate cancer: results from the FLAME randomized phase III trial. J Clin Oncol.

[4] Dearnaley D., Syndikus I., Mossop H., Khoo V., Birtle A., Bloomfield D. (2016). Conventional versus hypofractionated high-dose intensity-modulated radiotherapy for prostate cancer: 5-year outcomes of the randomised, non-inferiority, phase 3 CHHiP trial. Lancet Oncol.

[5] Tijssen R.H.N., Philippens M.E.P., Paulson E.S., Glitzner M., Chugh B., Wetscherek A. (2019). MRI commissioning of 1.5T MR-linac systems – a multi-institutional study. Radiother Oncol.

[6] Archambault Y., Boylan C., Bullock D., Morgas T., Peltola J., Ruokokoski E. (2020). Making on-line adaptive radiotherapy possible using artificial intelligence and machine learning for efficient daily re-planning. MPI.

[7] Fink C.A., Buchele C., Baumann L., Liermann J., Hoegen P., Ristau J. (2024). Dosimetric benefit of online treatment plan adaptation in stereotactic ultrahypofractionated MR-guided radiotherapy for localized prostate cancer. Front Oncol.

[8] Fink C.A., Ristau J., Buchele C., Klüter S., Liermann J., Hoegen-Saßmannshausen P. (2024). Stereotactic ultrahypofractionated MR-guided radiotherapy for localized prostate cancer - acute toxicity and patient-reported outcomes in the prospective, multicenter SMILE phase II trial. Clin Transl Radiat Oncol.

[9] Moazzezi M., Rose B., Kisling K., Moore K.L., Ray X. (2021). Prospects for daily online adaptive radiotherapy via ethos for prostate cancer patients without nodal involvement using unedited CBCT auto-segmentation. J Appl Clin Med Phys.

[10] Byrne M., Archibald-Heeren B., Hu Y., Teh A., Beserminji R., Cai E. (2022). Varian ethos online adaptive radiotherapy for prostate cancer: early results of contouring accuracy, treatment plan quality, and treatment time. J Appl Clin Med Phys.

[11] Waters M., Price A., Laugeman E., Henke L., Hugo G., Stowe H. (2024). CT-based online adaptive radiotherapy improves target coverage and organ at risk (OAR) avoidance in stereotactic body radiation therapy (SBRT) for prostate cancer. Clin Transl Radiat Oncol.

[12] Christiansen R.L., Dysager L., Hansen C.R., Jensen H.R., Schytte T., Nyborg C.J. (2022). Online adaptive radiotherapy potentially reduces toxicity for high-risk prostate cancer treatment. Radiother Oncol: J Eur Soc Ther Radiol Oncol.

[13] Westley R.L., Alexander S.E., Goodwin E., Dunlop A., Nill S., Oelfke U. (2024). Magnetic resonance image-guided adaptive radiotherapy enables safe CTV-to-PTV margin reduction in prostate cancer: a cine MRI motion study. Front Oncol.

[14] Byrne M., Teh A.Y.M., Archibald-Heeren B., Hu Y., Rijken J., Luo S. (2024). Intrafraction motion and margin assessment for ethos online adaptive radiotherapy treatments of the prostate and seminal vesicles. Adv Radiat Oncol.

[15] Sritharan K., Daamen L., Pathmanathan A., Schytte T., Pos F., Choudhury A. (2024). MRI-guided radiotherapy in twenty fractions for localised prostate cancer; results from the MOMENTUM study. Clin Transl Radiat Oncol.

[16] Schaeffer EM, Srinivas N, Ahmed B, An Y, Bitting R, Chapin B, et al. NCCN clinical practice guidelines in oncology, prostate cancer. 2025.

[17] Kalinauskaite G., Künzel L.A., Kluge A., Rubarth K., Dannehl J., Höhne C. (2025). Optimizing workflow for cone beam computed tomography-based online adaptive radiation therapy toward reduced physician involvement. Adv Radiat Oncol.

[18] Barnes H., Alexander S., Bower L., Ehlers J., Gani C., Herbert T. (2021). Development and results of a patient-reported treatment experience questionnaire on a 1.5 T MR-linac. Clin Transl. Radiat Oncol.

[19] Sand M, Wei J, Litwi M. Scoring Instructions for the E xpanded Prostate cancer Index Composite (EPIC) [Internet]. [cited 2025 Nov 5]. Available from: https://www.dropbox.com/scl/fi/0qrvnygicl0bzg1k3zov9/Urology-EPICScoringUtilitiesScoringDocumentation.pdf?rlkey=4vwvcze42e6vxlpe7loc38em4&e=1&st=do2s9gp5&dl=0.

[20] EORTC QLQ-C30 Scoring Manual [Internet]. EORTC; 2001 [cited 2025 Feb 27]. Available from: https://www.eortc.org/app/uploads/sites/2/2018/02/SCmanual.pdf.

[21] Skolarus T.A., Dunn R.L., Sanda M.G., Chang P., Greenfield T.K., Litwin M.S. (2015). Minimally important difference for the expanded prostate cancer index composite short form. Urology.

[22] Houben J., McColl G., Ham Kaanders J., Smeenk R.J. (2021). Patient reported toxicity and quality of life after hypofractionated high-dose intensity-modulated radiotherapy for intermediate- and high risk prostate cancer. Clin Transl Radiat Oncol.

[23] Staffurth J.N., Haviland J.S., Wilkins A., Syndikus I., Khoo V., Bloomfield D. (2021). Impact of hypofractionated radiotherapy on patient-reported outcomes in prostate cancer: results up to 5 yr in the CHHiP trial (CRUK/06/016). Eur Urol Oncol.

[24] Hall W.A., Raaijmakers F., Tree A., Christodouleas J.P., Der Voort V., vanZyp J. (2025). Daily online adaptive recontouring for prostate cancer using 1.5 tesla magnetic resonance image guidance (MRgRT) improves patient reported urinary symptoms, a prospective, international, observational cohort study (NCT04075305). Int J Radiat Oncol*Biol*Phys.

[25] Chavarriaga J. ASTRO 2025: Daily Online Adaptive Recontouring for Prostate Cancer Using 1.5 Tesla Magnetic Resonance Image Guidance (MRgRT) Improves Patient Reported Urinary Symptoms, A Prospective, International, Observational Cohort Study (NCT04075305). UroToday [Internet]. 2025 Sept 30 [cited 2025 Oct 20]; Available from: https://www.urotoday.com/conference-highlights/astro-2025/astro-2025-prostate-cancer/163503-astro-2025-daily-online-adaptive-recontouring-for-prostate-cancer-using-1-5-tesla-magnetic-resonance-image-guidance-mrgrt-improves-patient-reported-urinary-symptoms-a-prospective-international-observational-cohort-study-nct04075305.html.

[26] Zhu L.L., Bredfeldt J.S., Hu Y.-H., Hancox C., Guthier C.V., Quirk S. (2025). CT online adaptive radiotherapy is associated with dosimetric and acute toxicity improvements in prostate cancer treatment. Radiother Oncol.

