# Supplementary figures and images for: Prospective controlled study comparing patient-reported outcomes after daily online adaptive radiotherapy or conventional IGRT in patients with prostate cancer
Source: Clin Transl Radiat Oncol. 2025 Dec 5;57:101092. doi: 10.1016/j.ctro.2025.101092 (PMC12765114; doi:10.1016/j.ctro.2025.101092)

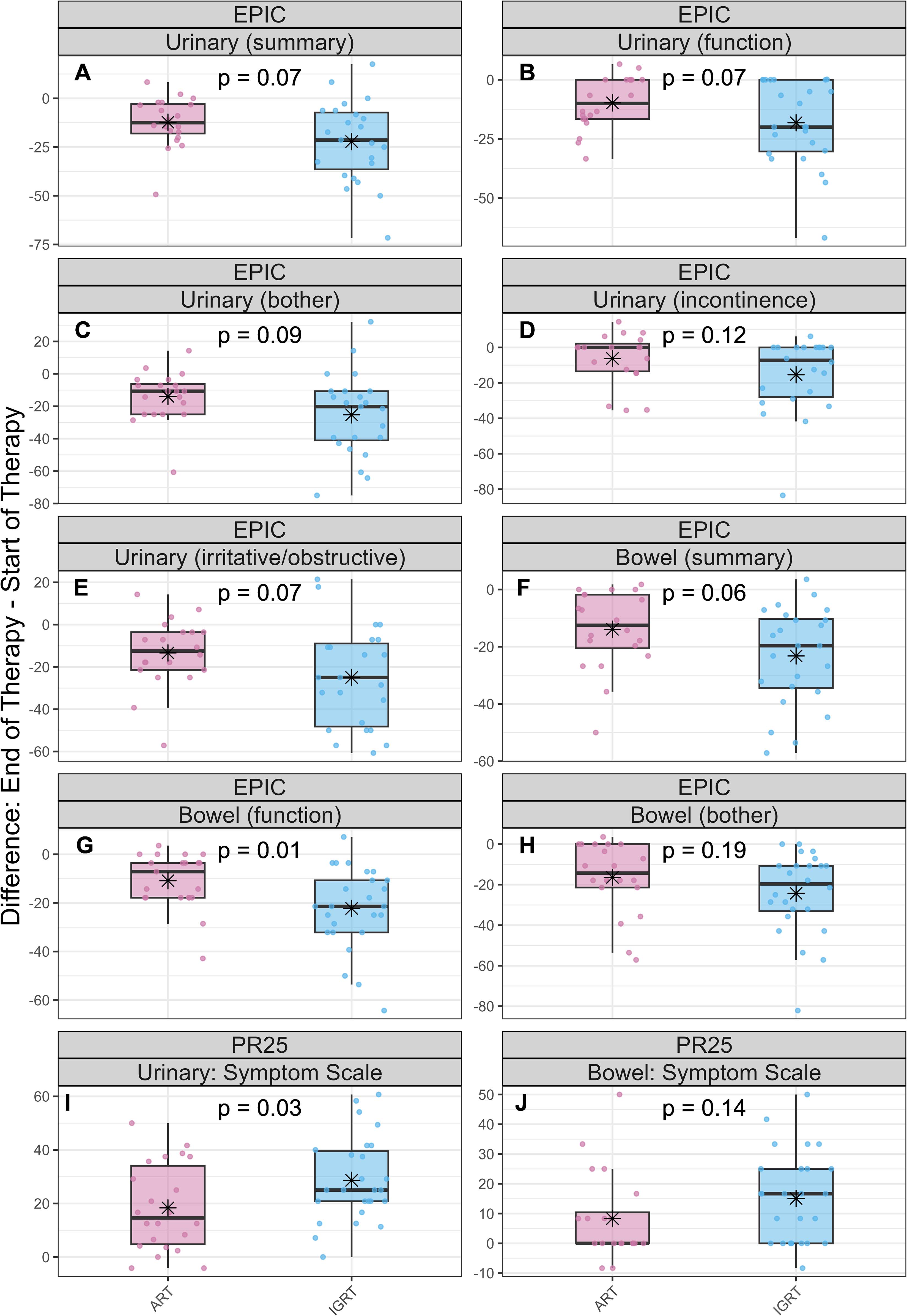

Supplement: Supplementary Fig. S1 — Changes in patient-reported urinary and bowel outcomes (oART vs. IGRT) analyzed in the subgroup without high/very-high NCCN risk. Boxplots showing changes from baseline to end of therapy in urinary and bowel domains, assessed by EPIC (A–H) and PR25 (I, J). Light Red = oART; light blue = IGRT. Black asterisks represent group means. p-values for t-test. A higher EPIC score indicates a better outcome, whereas a lower PR25 score indicates a better outcome. [file mmc1.jpg]

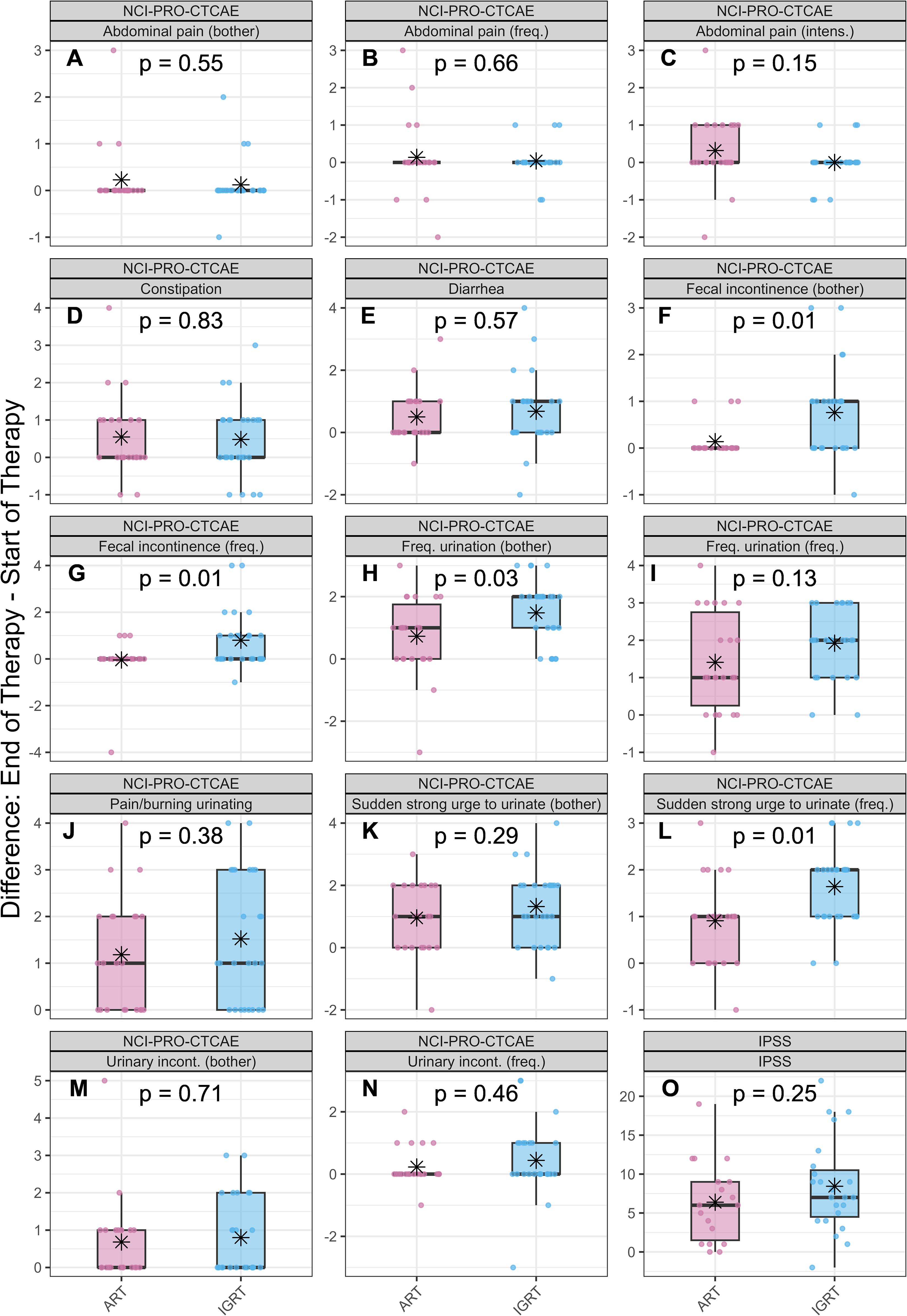

Supplement: Supplementary Fig. S2 — Changes in patient-reported acute toxicity (oART vs. IGRT) analyzed in the subgroup without high/very-high NCCN risk. Boxplots showing changes from baseline to end of therapy in different domains, assessed by NCI-PRO-CTCAE (A–N) and IPSS (O). Light Red = oART; light blue = IGRT. Black asterisks represent group means. p-values for t-test. Lower scores on both the NCI PRO CTCAE and the IPSS indicate better outcomes. [file mmc2.jpg]
