# Supplementary material for: Prospective controlled study comparing patient-reported outcomes after daily online adaptive radiotherapy or conventional IGRT in patients with prostate cancer
Source: Clin Transl Radiat Oncol. 2025 Dec 5;57:101092. doi: 10.1016/j.ctro.2025.101092 (PMC12765114; doi:10.1016/j.ctro.2025.101092)
Supplement: Supplementary Data 1 [file mmc3.docx]

| Table S1. Dose–Volume Objectives for Targets and Organs at Risk | | |
| --- | --- | --- |
|  | **Structure** | **Clinical goal (deviation)** |
| Priority 1 | Anorectum | D0.03cm³ ≤ 61.5 Gy (62.0 Gy) |
|  | PTV_62 GY | D98% ≥ 96% (95%) |
|  | PTV_48 GY | D98% ≥ 96% (95%) |
|  | PTV_57.6 GY | D98% ≥ 96% (95%) |
|  | PTV_62 GY | D2% ≤ 103% (105%) |
| Priority 2 | Anorectum | V50 Gy ≤ 10% (22%) |
|  | Anorectum | V40 Gy ≤ 22% (38%) |
|  | Bladder | V62 Gy ≤ 3% (5%) |
|  | Anorectum | V20G y ≤ 40% (85%) |
|  | Bladder | V48 Gy ≤ 15% (25%) |
|  | Bladder | V40 Gy ≤ 20% (50%) |
|  | Bag_Bowel | D0.03cm³ < 44 Gy (<= 46 Gy) |
| Priority 3 | PenileBulb | V40 Gy ≤ 30% (50%) |
|  | Anorectum | V30 Gy ≤ 30% (60%) |
|  | Skin | D0.5 cm³ ≤ 30.0 Gy (35.0 Gy) |
|  | RectalWall | D2% ≤ 30.0 Gy (35.0 Gy) |
| Priority 4 | Colon_sigmoid | V62 Gy ≤ 0.1% |
|  |  | V40 Gy ≤ 16% |
|  |  | V20 Gy ≤ 32% (70%) |
|  | Femur_Head_L/R | V50 Gy ≤ 5% (10%) |
|  | PenileBulb | D_Mean_ ≤ 20 Gy |
| Clinical planning goals (with acceptable deviations in parentheses) are listed by priority level for target volumes (PTV_62, PTV_57.6, PTV_48) and organs at risk. | | |

| Table S2. NCI PRO-CTCAE Item Descriptions for Symptom Questions (q1–q17) | |
| --- | --- |
| Question |  |
| q1 (a) | In the last 7 days, what was the SEVERITY of your DECREASED APPETITE at its WORST? |
| q1 (b) | In the last 7 days, how much did DECREASED APPETITE INTERFERE with your usual or daily activities? |
| q2 | In the last 7 days, did you have any INCREASED PASSING OF GAS (FLATULENCE)? |
| q3 | In the last 7 days, what was the SEVERITY of your CONSTIPATION at its WORST? |
| q4 | In the last 7 days, how OFTEN did you have LOOSE OR WATERY STOOLS (DIARRHEA/DIARRHOEA)? |
| q5 (a) | In the last 7 days, how OFTEN did you have PAIN IN THE ABDOMEN (BELLY AREA)? |
| q5 (b) | In the last 7 days, what was the SEVERITY of your PAIN IN THE ABDOMEN (BELLY AREA) at its WORST? |
| q5 (c) | In the last 7 days, how much did PAIN IN THE ABDOMEN (BELLY AREA) INTERFERE with your usual or daily activities? |
| q6 (a) | In the last 7 days, how OFTEN did you LOSE CONTROL OF BOWEL MOVEMENTS? |
| q6 (b) | In the last 7 days, how much did LOSS OF CONTROL OF BOWEL MOVEMENTS INTERFERE with your usual or daily activities? |
| q7 | In the last 7 days, what was the SEVERITY of your SKIN BURNS FROM RADIATION at their WORST? |
| q8 (a) | In the last 7 days, how OFTEN did you have PAIN? |
| q8 (b) | In the last 7 days, what was the SEVERITY of your PAIN at its WORST? |
| q8 (c) | In the last 7 days, how much did PAIN INTERFERE with your usual or daily activities? |
| q9 (a) | In the last 7 days, what was the SEVERITY of your INSOMNIA (INCLUDING DIFFICULTY FALLING ASLEEP, STAYING ASLEEP, OR WAKING UP EARLY) at its WORST? |
| q9 (b) | In the last 7 days, how much did INSOMNIA (INCLUDING DIFFICULTY FALLING ASLEEP, STAYING ASLEEP, OR WAKING UP EARLY) INTERFERE with your usual or daily activities? |
| q10 (a) | In the last 7 days, what was the SEVERITY of your FATIGUE, TIREDNESS, OR LACK OF ENERGY at its WORST? |
| q10 (b) | In the last 7 days, how much did FATIGUE, TIREDNESS, OR LACK OF ENERGY INTERFERE with your usual or daily activities? |
| q11 (a) | In the last 7 days, how OFTEN did you feel ANXIETY? |
| q11 (b) | In the last 7 days, what was the SEVERITY of your ANXIETY at its WORST? |
| q11 (c) | In the last 7 days, how much did ANXIETY INTERFERE with your usual or daily activities? |
| q12 | In the last 7 days, what was the SEVERITY of your PAIN OR BURNING WITH URINATION at its WORST? |
| q13 (a) | In the last 7 days, how OFTEN did you feel an URGE TO URINATE ALL OF A SUDDEN? |
| q13 (b) | In the last 7 days, how much did SUDDEN URGES TO URINATE INTERFERE with your usual or daily activities? |
| q14 (a) | In the last 7 days, were there times when you had to URINATE FREQUENTLY? |
| q14 (b) | In the last 7 days, how much did FREQUENT URINATION INTERFERE with your usual or daily activities? |
| q15 (a) | In the last 7 days, how OFTEN did you have LOSS OF CONTROL OF URINE (LEAKAGE)? |
| q15 (b) | In the last 7 days, how much did LOSS OF CONTROL OF URINE (LEAKAGE) INTERFERE with your usual or daily activities? |
| q16 | In the last 7 days, what was the SEVERITY of your DIFFICULTY GETTING OR KEEPING AN ERECTION at its WORST? |
| q17 | Do you have any other symptoms that you wish to report? |
| Abbreviations: NCI PRO-CTCAE = National Cancer Institute’s Patient-Reported Outcomes version of the Common Terminology Criteria for Adverse Events | |

| Table S3a. Mean EPIC Baseline Scores in oART versus IGRT groups | | | |
| --- | --- | --- | --- |
| Parameter | **oART (mean (SD))** | **IGRT (mean (SD))** | **p-value** |
| Urinary summary | 86.76 (8.85) | 87.95 (15.26) | 0.696 |
| Urinary function | 95.72 (5.40) | 94.50 (9.95) | 0.533 |
| Urinary bother | 80.36 (12.73) | 83.12 (20.09) | 0.505 |
| Urinary incontinence | 94.65 (7.75) | 91.04 (15.24) | 0.221 |
| Urinary irritative/obstructive | 83.53 (12.35) | 86.48 (17.80) | 0.437 |
| Bowel summary | 95.61 (5.69) | 92.98 (12.77) | 0.274 |
| Bowel function | 94.98 (6.65) | 94.33 (10.21) | 0.758 |
| Bowel bother | 95.82 (6.53) | 91.71 (15.71) | 0.160 |
| Sexual summary | 33.43 (25.11) | 43.27 (25.02) | 0.133 |
| Sexual function | 26.71 (25.96) | 33.00 (27.79) | 0.359 |
| Sexual bother | 50.00 (34.47) | 62.26 (32.48) | 0.167 |
| Hormone summary | 84.63 (13.92) | 85.79 (15.48) | 0.760 |
| Hormone function | 81.91 (17.58) | 82.41 (19.85) | 0.917 |
| Hormone bother | 87.31 (11.61) | 88.58 (13.31) | 0.691 |
| Abbreviations: EPIC = Expanded Prostate Cancer Index Composite; oART = online adaptive radiotherapy; IGRT = image-guided radiotherapy; SD = standard deviation. | | | |

| Table S3b. Mean PR25 Baseline Scores in oART versus IGRT groups | | | |
| --- | --- | --- | --- |
| Parameter | o**ART (mean (SD))** | **IGRT (mean (SD))** | **p-value** |
| Symptom_Scale_URI | 20.68 (15.43) | 20.07 (16.87) | 0.880 |
| Symptom_Scale_AID | 8.33 (16.67) | NaN (NA) | NA |
| Symptom_Scale_BOW | 3.01 (5.69) | 5.17 (10.31) | 0.287 |
| Symptom_Scale_HTR | 12.04 (10.08) | 9.68 (12.65) | 0.401 |
| Functional_Scale_SAC | 74.29 (21.52) | 65.48 (26.42) | 0.150 |
| Functional_Scale_SFU | 59.90 (16.16) | 62.41 (14.92) | 0.657 |
| Abbreviations: AID = incontinence aid; BOW = bowel symptoms; IGRT = image-guided radiotherapy; HTR = Hormonal Treatment-Related Symptoms; oART = online adaptive radiotherapy; SAC = sexual activity; SD = standard deviation; SFU = sexual functioning; URI – urinary symptoms; | | | |

| Table S3c. Mean IPSS Baseline Scores in oART versus IGRT groups | | | |
| --- | --- | --- | --- |
| Stratified by group | **oART** | **IGRT** | **p-value** |
| Symptom Severity (%) |  |  | 0.612 |
| Mild symptoms (0 – 7) | 12 (36.4%) | 9 (36.0%) |  |
| Moderate symptoms (8 – 19) | 14 (42.4%) | 13 (52.0%) |  |
| Severe symptoms (20 – 35) | 7 (21.2%) | 3 (12.0%) |  |
| IPSS baseline score (mean (SD)) | 11.52 (7.08) | 11.24 (8.40) | 0.893 |
| Abbreviations: IGRT = image-guided radiotherapy; IPSS = International Prostate Symptom Score; oART = online adaptive radiotherapy; SD = Standard Deviation | | | |

| Table S4. Dosimetric Parameters for OAR in Reference Plans by Treatment Group | | | |
| --- | --- | --- | --- |
| Parameter | **ART (mean (SD))** | **IGRT (mean (SD))** | **p-value** |
| Rectum V62.0 | 0.03 (0.06) | 0.01 (0.02) | 0.321 |
| Rectum d0.03 | 61.56 (0.38) | 61.54 (0.28) | 0.915 |
| RectumV50 Gy | 15.16 (5.45) | 14.70 (5.14) | 0.717 |
| Rectum V40 Gy | 21.79 (5.21) | 22.57 (4.84) | 0.517 |
| Rectum V20 Gy | 45.47 (12.45) | 47.27 (12.96) | 0.550 |
| Rectum V30 Gy | 30.67 (6.57) | 32.94 (7.87) | 0.183 |
| Bladder V62 Gy | 2.21 (5.06) | 2.45 (5.26) | 0.844 |
| Bladder V48 Gy | 15.19 (6.30) | 15.53 (6.30) | 0.820 |
| Bladder V40 Gy | 20.11 (8.88) | 18.81 (8.91) | 0.540 |
| Penil Bulb V40 Gy | 35.51 (21.48) | 38.65 (20.78) | 0.534 |
| Penil Bulb mean | 32.17 (16.47) | 35.61 (19.48) | 0.417 |
| Abbreviations: OAR = organs at risk; oART = online adaptive radiotherapy; IGRT = image-guided radiotherapy; SD = standard deviation. | | | |

| Table S5. Mean difference end-of-therapy to baseline EPIC scores in oART versus IGRT groups | | | |
| --- | --- | --- | --- |
| Subscale | **ART (Mean (SD))** | **IGRT (Mean (SD))** | **p-value** |
| Urinary summary | -12.15 (15.60) | -20.57 (20.50) | 0.069 |
| Urinary function | -9.53 (14.71) | -17.47 (17.33) | 0.052 |
| Urinary bother | -14.00 (19.01) | -22.97 (25.28) | 0.113 |
| Urinary incontinence | -5.47 (15.18) | -13.93 (20.18) | 0.066 |
| Urinary irritative/obstructive | -13.79 (20.84) | -23.28 (24.01) | 0.099 |
| Bowel summary | -15.13 (17.23) | -20.41 (17.36) | 0.233 |
| Bowel function | -12.64 (17.20) | -19.70 (17.14) | 0.102 |
| Bowel bother | -16.70 (19.97) | -21.30 (20.42) | 0.372 |
| Sexual summary | -4.16 (10.62) | -13.76 (20.55) | 0.038 |
| Sexual function | -6.51 (12.04) | -11.27 (18.91) | 0.259 |
| Sexual bother | 1.16 (19.46) | -21.20 (44.22) | 0.022 |
| Hormone summary | 1.12 (12.88) | -7.97 (10.67) | 0.007 |
| Hormone function | 0.31 (17.04) | -7.10 (13.65) | 0.081 |
| Hormone bother | 1.74 (11.21) | -8.10 (13.57) | 0.004 |
| Abbreviations: EPIC = Expanded Prostate Cancer Index Composite; oART = online adaptive radiotherapy; IGRT = image-guided radiotherapy; SD = standard deviation. | | | |

| Table S6. Baseline and end-of-therapy EPIC domain scores for oART versus IGRT groups (Mean [SD]) | | | | | | |
| --- | --- | --- | --- | --- | --- | --- |
|  | **Baseline** | | | **End of RT** | | |
| Subscale | **oART**  **(n=37)** | **IGRT**  **(n=29)** | **p-value** | **oART**  **(n=37)** | **IGRT**  **(n=29)** | **p-value** |
| Urinary summary | 86.76 (8.85) | 87.95 (15.26) | 0.696 | 75.30 (16.13) | 67.13 (22.02) | 0.089 |
| Urinary function | 95.72 (5.40) | 94.50 (9.95) | 0.533 | 86.56 (15.31) | 76.83 (20.62) | 0.033 |
| Urinary bother | 80.36 (12.73) | 83.12 (20.09) | 0.505 | 67.26 (19.22) | 60.33 (24.77) | 0.208 |
| Urinary incontinence | 94.65 (7.75) | 91.04 (15.24) | 0.221 | 89.33 (17.54) | 77.44 (27.06) | 0.040 |
| Urinary irritative/  obstructive | 83.53 (12.35) | 86.48 (17.80) | 0.437 | 70.56 (19.44) | 63.39 (23.88) | 0.187 |
| Bowel summary | 95.61 (5.69) | 92.98 (12.77) | 0.274 | 80.65 (16.84) | 72.78 (21.63) | 0.104 |
| Bowel function | 94.98 (6.65) | 94.33 (10.21) | 0.758 | 82.34 (15.84) | 74.63 (20.05) | 0.086 |
| Bowel bother | 95.82 (6.53) | 91.71 (15.71) | 0.160 | 79.38 (20.51) | 70.94 (25.48) | 0.144 |
| Sexual summary | 33.43 (25.11) | 43.27 (25.02) | 0.133 | 28.27 (23.88) | 30.39 (28.19) | 0.766 |
| Sexual function | 26.71 (25.96) | 33.00 (27.79) | 0.359 | 19.00 (23.18) | 21.83 (27.42) | 0.672 |
| Sexual bother | 50.00 (34.47) | 62.26 (32.48) | 0.167 | 47.63 (35.29) | 45.50 (40.16) | 0.837 |
| Hormone summary | 84.63 (13.91) | 85.79 (15.48) | 0.760 | 87.13 (9.21) | 77.71 (16.61) | 0.006 |
| Hormone function | 81.91 (17.58) | 82.41 (19.85) | 0.917 | 83.71 (12.27) | 75.00 (18.22) | 0.029 |
| Hormone bother | 87.31 (11.68) | 88.58 (13.31) | 0.691 | 90.02 (8.62) | 80.62 (17.22) | 0.007 |
| Abbreviations: EPIC = Expanded Prostate Cancer Index Composite; oART = online adaptive radiotherapy; IGRT = image-guided radiotherapy; SD = standard deviation. | | | | | | |

| Table S7. Mean (SD) difference end-of-therapy to baseline EORTC QLQ-PR25 scores in oART versus IGRT groups | | | |
| --- | --- | --- | --- |
| Subscale | **ART (Mean (SD))** | **IGRT (Mean (SD))** | **p-value** |
| Urinary | 20.0 (16.0) | 27.5 (16.1) | 0.064 |
| Incontinence Aid | 11.1 (38.5) | NA (NA) | NA |
| Bowel | 10.5 (16.7) | 14.1 (15.4) | 0.374 |
| HTR | -1.10 (9.58) | 2.74 (11.2) | 0.147 |
| Sexual activity | 11.8 (18.1) | 15.5 (20.8) | 0.462 |
| Sexual function | 4.63 (20.0) | -2.50 (20.0) | 0.450 |
| Abbreviations: EORTC QLQ-PR25 = European Organisation for Research and Treatment of Cancer Quality of Life Questionnaire–Prostate Cancer module; oART = online adaptive radiotherapy; IGRT = image-guided radiotherapy; HTR = hormonal treatment–related symptoms; NA = not applicable. | | | |

| Table S8. Mean (SD) difference end-of-therapy to baseline NCI PRO-CTCAE symptom scores in oART versus IGRT groups | | | |
| --- | --- | --- | --- |
| Question* | **ART (Mean (SD))** | **IGRT (Mean (SD))** | **p-value** |
| q1 (a) | 0.22 (0.53) | 0.04 (0.92) | 0.325 |
| q1 (b) | 0.16 (0.50) | 0.00 (0.61) | 0.243 |
| q2 | 0.30 (0.66) | 0.36 (0.56) | 0.701 |
| q3 | 0.70 (1.20) | 0.50 (1.00) | 0.472 |
| q4 | 0.62 (1.11) | 0.61 (1.17) | 0.960 |
| q5 (a) | 0.27 (1.04) | -0.04 (0.58) | 0.168 |
| q5 (b) | 0.38 (1.04) | -0.07 (0.60) | 0.045 |
| q5 (c) | 0.27 (0.77) | 0.11 (0.50) | 0.332 |
| q6 (a) | 0.03 (1.12) | 0.71 (1.18) | 0.020 |
| q6 (b) | 0.27 (0.77) | 0.68 (0.94) | 0.059 |
| q7 | -0.08 (0.49) | -0.14 (0.76) | 0.692 |
| q8 (a) | 0.43 (1.07) | 0.50 (1.07) | 0.802 |
| q8 (b) | 0.51 (1.12) | 0.68 (1.31) | 0.586 |
| q8 (c) | 0.41 (1.07) | 0.61 (1.26) | 0.487 |
| q9 (a) | 0.65 (1.27) | 0.86 (1.35) | 0.527 |
| q9 (b) | 0.59 (0.96) | 0.71 (0.98) | 0.622 |
| q10 (a) | 0.54 (1.10) | 0.71 (1.12) | 0.532 |
| q10 (b) | 0.30 (0.97) | 0.50 (1.07) | 0.428 |
| q11 (a) | 0.08 (0.64) | -0.04 (0.74) | 0.500 |
| q11 (b) | 0.08 (0.64) | 0.04 (1.04) | 0.829 |
| q11 (c) | 0.08 (0.43) | 0.32 (0.77) | 0.116 |
| q12 | 1.32 (1.31) | 1.46 (1.35) | 0.675 |
| q13 (a) | 0.95 (1.20) | 1.57 (0.88) | 0.023 |
| q13 (b) | 0.97 (1.17) | 1.32 (1.16) | 0.236 |
| q14 (a) | 1.43 (1.28) | 1.79 (1.10) | 0.247 |
| q14 (b) | 0.78 (1.23) | 1.36 (1.13) | 0.058 |
| q15 (a) | 0.19 (0.62) | 0.32 (1.22) | 0.570 |
| q15 (b) | 0.46 (0.99) | 0.71 (1.01) | 0.313 |
| q16 | 0.86 (1.51) | 0.82 (1.42) | 0.907 |
| q17 | 0.14 (0.54) | 0.18 (0.55) | 0.750 |
| Abbreviations: NCI PRO-CTCAE = National Cancer Institute’s Patient-Reported Outcomes version of the Common Terminology Criteria for Adverse Events; oART = online adaptive radiotherapy; IGRT = image-guided radiotherapy.  *Questions (q1–q17) correspond to specific NCI PRO-CTCAE symptom items; full item descriptions are provided in Supplementary Table S6 | | | |

| Table S9. Mean (SD) difference end-of-therapy to baseline IPSS scores in oART versus IGRT groups | | | |
| --- | --- | --- | --- |
|  | **ART (Mean (SD))** | **IGRT (Mean (SD))** | **p-value** |
| IPSS | 6.55 (6.60) | 8.64 (6.22) | 0.22 |
| Abbreviations: IPSS = International Prostate Symptom Score; oART = online adaptive radiotherapy; IGRT = image-guided radiotherapy. | | | |

| Table S10. Patient characteristics for cohort without high/very (NCCN) high risk groups | | | |
| --- | --- | --- | --- |
| Characteristic | **ART (N=25)** | **IGRT (N=28)** | **p-value** |
| Age, mean (SD) | 70.48 (7.51) | 72.54 (6.95) | 0.305 |
| Initial PSA, mean (SD) | 7.81 (3.23 | 8.41 (4.73) | 0.601 |
| NCCN risk group (%) |  |  | 0.750 |
| Low/Very low | 3 (12.0) | 4 (14.3) |  |
| Favourable intermediate | 17 (68.0) | 16 (57.1) |  |
| Unfavourable intermediate | 5 (20.0) | 8 (28.6) |  |
| Androgen deprivation therapy (%) | 5 (20) | 7 (25.0) | 0.916 |
| Abbreviations: ADT = androgen deprivation therapy; ART = adaptive radiotherapy; CTV = clinical target volume; IGRT = image-guided radiotherapy; IQR = interquartile range; N.A. = not applicable; NCCN = National Comprehensive Cancer Network; PSA = prostate-specific antigen; SD = standard deviation | | | |

| Table S11. Mean difference end-of-therapy to baseline EPIC scores in oART versus IGRT groups (subgroup without high/very-high NCCN risk) | | | |
| --- | --- | --- | --- |
| Subscale | **ART (Mean (SD))** | **IGRT (Mean (SD))** | **p-value** |
| Urinary summary | -12.24 (12.77) | -12.24 (12.77) | 0.072 |
| Urinary function | -9.74 (11.23) | -18.17 (17.73) | 0.073 |
| Urinary bother | -13.93 (15.54 | -25.26 (25.60) | 0.093 |
| Urinary incontinence | -6.24 (14.83) | -15.43 (21.24) | 0.122 |
| Urinary irritative/obstructive | -13.39 (16.34) | -25.00 (23.76) | 0.073 |
| Bowel summary | -13.90 (13.77) | -23.21 (17.10 | 0.056 |
| Bowel function | -10.88 (11.43) | -22.29 (17.01) | 0.012 |
| Bowel bother | -16.43 (17.85) | -24.26 (20.50) | 0.189 |
| Sexual summary | -5.43 (10.57) | -15.42 (21.17) | 0.086 |
| Sexual function | -5.88 (12.75) | -14.41 (18.81) | 0.117 |
| Sexual bother | -4.41 (16.79) | -19.08 (44.61) | 0.211 |
| Hormone summary | -5.83 (9.77) | -10.02 (9.25) | 0.183 |
| Hormone function | -8.06 (13.95) | -8.69 (13.08) | 0.884 |
| Hormone bother | -3.68 (9.43) | -3.68 (9.43) | 0.060 |
| Abbreviations: EPIC = Expanded Prostate Cancer Index Composite; oART = online adaptive radiotherapy; IGRT = image-guided radiotherapy; SD = standard deviation. | | | |

| Table S12. Mean (SD) difference end-of-therapy to baseline EORTC QLQ-PR25 scores in oART versus IGRT groups (subgroup without high/very-high NCCN risk) | | | |
| --- | --- | --- | --- |
| Subscale | **ART (Mean (SD))** | **IGRT (Mean (SD))** | **p-value** |
| Urinary | 18.3 (16.2) | 28.6 (15.8) | 0.032 |
| Incontinence Aid | 33.3 (NA) | NA (NA) | NA |
| Bowel | 8.33 (14.8) | 15.1 (15.3) | 0.139 |
| HTR | -0.343 (8.99) | 4.76 (9.25) | 0.063 |
| Sexual activity | 10.5 (15.9) | 18.0 (19.2) | 0.166 |
| Sexual function | 3.57 (23.0) | -2.78 (21.2) | 0.581 |
| Abbreviations: EORTC QLQ-PR25 = European Organisation for Research and Treatment of Cancer Quality of Life Questionnaire–Prostate Cancer module; oART = online adaptive radiotherapy; IGRT = image-guided radiotherapy; HTR = hormonal treatment–related symptoms; NA = not applicable. | | | |

| Table S13. Mean (SD) difference end-of-therapy to baseline NCI PRO-CTCAE symptom scores in oART versus IGRT (subgroup without high/very-high NCCN risk) | | | |
| --- | --- | --- | --- |
| Question* | **ART (Mean (SD))** | **IGRT (Mean (SD))** | **p-value** |
| q1 (a) | 0.32 (0.65) | 0.04 (0.98) | 0.263 |
| q1 (b) | 0.27 (0.63) | 0.00 (0.65) | 0.151 |
| q2 | 0.14 (0.64) | 0.40 (0.58) | 0.144 |
| q3 | 0.55 (1.10) | 0.48 (1.00) | 0.832 |
| q4 | 0.50 (0.86) | 0.68 (1.22) | 0.566 |
| q5 (a) | 0.14 (0.99) | 0.04 (0.45) | 0.664 |
| q5 (b) | 0.32 (0.95) | 0.00 (0.50) | 0.149 |
| q5 (c) | 0.23 (0.69) | 0.12 (0.53) | 0.548 |
| q6 (a) | -0.05 (0.95) | 0.80 (1.22) | 0.012 |
| q6 (b) | 0.14 (0.35) | 0.76 (0.97) | 0.007 |
| q7 | -0.18 (0.59) | -0.16 (0.80) | 0.917 |
| q8 (a) | 0.27 (0.77) | 0.56 (1.12) | 0.317 |
| q8 (b) | 0.23 (0.75) | 0.76 (1.36) | 0.111 |
| q8 (c) | 0.14 (0.64) | 0.68 (1.31) | 0.085 |
| q9 (a) | 0.23 (1.11) | 0.80 (1.26) | 0.107 |
| q9 (b) | 0.32 (0.84) | 0.71 (0.98) | 0.622 |
| q10 (a) | 0.45 (1.01) | 0.80 (1.04) | 0.256 |
| q10 (b) | 0.23 (0.92) | 0.56 (1.08) | 0.266 |
| q11 (a) | -0.05 (0.58) | -0.08 (0.76) | 0.863 |
| q11 (b) | -0.05 (0.49) | 0.04 (1.10) | 0.738 |
| q11 (c) | 0.14 (0.47) | 0.36 (0.81) | 0.261 |
| q12 | 1.18 (1.22) | 1.52 (1.39) | 0.383 |
| q13 (a) | 0.91 (0.92) | 1.64 (0.86) | 0.007 |
| q13 (b) | 0.95 (1.13) | 1.32 (1.18) | 0.286 |
| q14 (a) | 1.41 (1.33) | 1.92 (0.91) | 0.128 |
| q14 (b) | 0.73 (1.28) | 1.48 (1.05) | 0.032 |
| q15 (a) | 0.23 (0.61) | 0.32 (1.22) | 0.455 |
| q15 (b) | 0.68 (1.13) | 0.80 (1.04) | 0.711 |
| q16 | 1.00 (1.80) | 0.68 (1.22) | 0.474 |
| q17 | 0.00 (0.44) | 0.20 (0.50) | 0.154 |
| Abbreviations: NCI PRO-CTCAE = National Cancer Institute’s Patient-Reported Outcomes version of the Common Terminology Criteria for Adverse Events; oART = online adaptive radiotherapy; IGRT = image-guided radiotherapy.  *Questions (q1–q17) correspond to specific NCI PRO-CTCAE symptom items; full item descriptions are provided in Supplementary Table S6 | | | |

| Table S14. Mean (SD) difference end-of-therapy to baseline IPSS scores in oART versus IGRT groups (subgroup without high/very-high NCCN risk) | | | |
| --- | --- | --- | --- |
|  | **ART (Mean (SD))** | **IGRT (Mean (SD))** | **p-value** |
| IPSS | 6.38 (5.19) | 8.43 (5.94) | 0.25 |
| Abbreviations: IPSS = International Prostate Symptom Score; oART = online adaptive radiotherapy; IGRT = image-guided radiotherapy. | | | |

| Table S15. Minimal clinically important differences for EPIC and PR-25 questionnaires (subgroup without high/very-high NCCN risk) | | | | |
| --- | --- | --- | --- | --- |
| PROM | **Subdomain** | **ART, n (%)** | **IGRT, n (%)** | **p-value** |
| PR-25 | Urinary | 14/22 (64%) | 24/26 (92%) | 0.037 |
|  | Bowel | 5/20 (25%) | 14/26 (54%) | 0.095 |
| EPIC | Urinary incontinence | 7/21 (37%) | 11/25 (50%) | 0.595 |
|  | Urinary irritative/obstructive | 13/21 (65%) | 19/25 (83%) | 0.332 |
|  | Bowel | 13/21 (65%) | 22/25 (92%) | 0.071 |
